# Biopsy or Biomarker? Children With Minimal Change Disease Have a Distinct Profile of Urinary Epidermal Growth Factor

**DOI:** 10.3389/fped.2021.727954

**Published:** 2021-11-25

**Authors:** Niels Lodeweyckx, Kristien Wouters, Kristien J. Ledeganck, Dominique Trouet

**Affiliations:** ^1^Department of Pediatric Nephrology, Antwerp University Hospital, Edegem, Belgium; ^2^Clinical Trial Center (CTC), Clinical Research Center (CRC) Antwerp, Antwerp University Hospital, University of Antwerp, Edegem, Belgium; ^3^Laboratory of Experimental Medicine and Pediatrics and Member of the Infla-Med Centre of Excellence, University of Antwerp, Edegem, Belgium

**Keywords:** minimal change disease, urinary epidermal growth factor, ACE-inhibition, diagnostic biomarker, genetic predisposition

## Abstract

**Background:** In this study, the profile of urinary EGF excretion (uEGF/uCreat) was mapped in children presenting with prolonged proteinuria or with nephrotic syndrome refractory to or dependent of steroids. We investigated whether uEGF/uCreat could be linked to the underlying biopsy result, taking into account its response to immunosuppressive medication and to ACE inhibition, as well as genetic predisposition.

**Methods:** Ninety-eight pediatric patients with initial presentation of nephrotic syndrome or prolonged proteinuria were included in this study, along with 49 healthy controls and 20 pediatric Alport patients. All patients had a normal kidney function and were normotensive during the course of the study, whether or not under ACE inhibition. In repeated urine samples, uEGF was measured and concentration was normalized by urine creatinine. In order to compare diagnosis on kidney biopsy, genetic predisposition and response of uEGF/uCreat to immunosuppression and to ACE inhibition, uEGF/uCreat is studied in a linear mixed effects model.

**Results:** Patients with Minimal Change Disease (MCD) showed a significantly different profile of uEGF/uCreat in comparison to healthy children, as well as compared to patients with Focal Segmental Glomerulosclerosis (FSGS) or another glomerulopathy on kidney biopsy. The response of uEGF/uCreat to ACE inhibition was absent in minimal change disease and contrasted with an impressive beneficial effect of ACE inhibition on uEGF/uCreat in FSGS and other proteinuric glomerulopathies. Absence of a genetic predisposition was also associated with a significantly lower uEGF/uCreat.

**Conclusions:** Despite preserved kidney function, children with a proteinuric or nephrotic glomerular disease on kidney biopsy show a significantly lower uEGF/uCreat, indicative of early tubulo-interstitial damage, which appears reversible under ACE inhibition in any underlying glomerulopathy except in minimal change disease. In view of the distinct profile of uEGF/uCreat in minimal change disease compared to other glomerulopathies, and the link between genetic predisposition and uEGF/uCreat, our study suggests that uEGF/uCreat can be a helpful tool to decide on the need for a renal biopsy in order to differentiate minimal change disease from other proteinuric glomerular diseases.

## Introduction

Children presenting with a first episode of nephrotic-range proteinuria without kidney failure are prompt treated with high-dose steroids, assuming an underlying minimal change disease (MCD) favorably responding to a single cure of steroids (1–3). However, when this idiopathic nephrotic syndrome acquires a frequent relapsing, steroid-dependent, or steroid-resistant character, the initiation of other immunosuppressive therapy is most often guided by the findings on kidney biopsy (4, 5). Intriguingly, in this subgroup of refractory nephrotic children, up to half of the biopsy results reveal normal findings on light microscopy with no evidence of glomerular disease on immunofluorescence (4, 6). Although ultrastructural abnormalities are not obligatory present, the effacement of the podocytes' foot processes on electron microscopy are the only hint to a suggested underlying podocyte disease called minimal change disease (7). Those minimal morphological features may even disappear after immunosuppressive treatment, and are indicative of a reversible fragility of the podocytes to exogenous factors in MCD. In contrast, the sclerotic lesions on light microscopy in Focal Segmental Glomerulosclerosis (FSGS) encountered in a minority of the nephrotic patients are irreversible and point out to a more severe, genetically driven podocyte disease translated into the clinical features of an often steroid-resistant nephrotic syndrome with high chance of evolution toward chronic kidney failure (8–10).

Despite the overlap in clinical presentation, and in spite of similar therapeutical responses on T-cell or B-cell inhibition, MCD is considered a more benign podocyte disease than FSGS, with a different underlying genetic predisposition and pathophysiology (7). Of course, it should be kept in mind that the limited number of glomeruli within one biopsy sample could falsely identify an intrinsic FSGS as MCD, sometimes requiring repeated biopsies to unmask a “fake” MCD (7, 8). Moreover, recent research in adults provides increasing evidence that MCD is a distinct independent podocyte disease.

Comparative differential proteomic analyses recently gave evidence for a different profile of urinary biomarkers in MCD compared to FSGS (11).

In case of prolonged unexplained non-nephrotic proteinuria, either or not associated with hematuria or hypertension, children are also referred for a kidney biopsy to reveal the underlying glomerulopathy, in order to start tailored immunosuppressive treatment (12). In this group of pediatric patients besides MCD or FSGS, kidney biopsy often reveals an underlying IgA nephropathy, and less frequently C3 glomerulopathy, membranous glomerulonephritis, or other glomerulopathies rarely encountered at pediatric age (13, 14). The degree of genetic predisposition in this group of patients still remains unclear (15).

Due to its unique origin, mostly restricted to kidney tissue (16), urinary Epidermal Growth Factor (uEGF) functions as a predictive marker for progression in renal decline, and directly indicates the mitogenic function and interstitial regenerative capacity of the kidney (17–19). uEGF levels correlate with intrarenal mRNA expression of EGF on kidney biopsy and inversely correlate with the degree of interstitial fibrosis and tubular atrophy (16, 19, 20). Theoretically, already at an early stage in the disease, a decrease in uEGF could thus precede glomerular deterioration visualized on kidney biopsy (15, 19, 21). Tubular injury is closely linked to progression in glomerular nephropathies (22). Therefore, tubular proteins such as uEGF are potentially better predictive markers for progression of glomerular damage and renal deterioration than proteinuria and albuminuria (17). Particularly for uEGF, over the last few years, increasing evidence highlights its added value in predicting kidney deterioration in many disease entities such as systemic lupus disease (21), diabetes mellitus (23), but also in IgA nephropathy (15, 24), and other chronic kidney diseases (17) in adults. Specifically in children, recent reports illustrated the prognostic capacities of uEGF regarding renal decline in pediatric patients with chronic kidney disease (25), Alport syndrome (20), and nephrotic syndrome (19).

In this present study, we formulated three subsequent research questions. First, we explored whether uEGF could be linked to biopsy result (MCD or other proteinuric glomerulopathies) in children presenting with either prolonged non-nephrotic proteinuria or displaying a steroid-resistant or steroid-dependent nephrotic syndrome. Second, we investigated the impact of immunosuppression and ACE inhibition on the profile of uEGF in these patients. Third, we investigated if uEGF is related to a genetic predisposition of proteinuric glomerulopathies in this pediatric study population.

## Patients and Methods

### Study Design

In this longitudinal ambispective observational clinical trial, 98 pediatric patients with the initial presentation of nephrotic or non-nephrotic proteinuria were included between March 2016 and April 2021 at the Antwerp University Hospital. At three time points, with an interval of at least 1 month, urine samples were collected from each patient to determine creatinine, protein, and uEGF.

#### Study Patients

Prior to the design and initiation of this study, the patients had already undergone a renal needle biopsy at some point of the disease course, and were on a biopsy-guided immunosuppressive treatment at the start of the study. As a consequence, children with steroid-resistant, steroid-dependent, or frequent-relapsing nephrotic syndrome were included, as well as children without nephrotic syndrome but with unexplained prolonged proteinuria more than 0.5 g/g creatinine, either or not associated with hematuria.

In every patient presenting with hypertension or residual proteinuria after initiation of immunosuppression, ACE inhibition had been started, irrespective of and prior to the initiation of the study. Patients were in remission under treatment at the time the urine samples for the study were taken, thereby displaying not more than 0.3 g/g protein/creatinine in the urine.

Included patients had to be normotensive and have a normal kidney function [estimated glomerular filtration rate (eGFR) > 90 ml/min/1.73 m^2^] at the start of and during the course of the study.

Exclusion criteria were the use of diuretics or aminoglycosides, an active urinary tract infection, or severe co-morbidity.

#### Healthy Control Patients

A recently published healthy control group (*n* = 49) functioned as reference for normal, age-specific uEGF values (26). Another group of patients was also included as a second, separate control group, in order to determine the effect of ACE inhibition without immunosuppression on uEGF. These children had initially presented with isolated hematuria (persistent microscopic hematuria with or without intermittent macroscopic hematuria) (*n* = 20). Diagnosis of Alport syndrome in these patients had been made by genetic testing and/or by kidney biopsy. All but three patients were on preventive monotherapy with ACE inhibition as recommended (27), but were not treated with any immunosuppression. Every Alport patient was normotensive, had a normal kidney function, and showed no proteinuria under ACE inhibition during the course of the study.

#### Ethics

The study was conducted in accordance with the *Declaration of Helsinki* and the principles of Good Clinical Practice. The protocol was approved by the Ethics Committee of the Antwerp University Hospital (file number 9/44/231). All patients and their parents and/or legal guardians gave a written informed consent.

### Determination of Urinary EGF

Urinary EGF was measured using an EGF human Elisa kit (Invitrogen, Waltham, MA, USA) according to the manufacturer's guidelines. The detection limit of this assay was 3.9 pg/ml. The intra-and intervariability of the EGF human Elisa kit was excellent (28).

In order to avoid bias from differences in urinary concentration, uEGF was expressed as uEGF/U creatinine ratio. In view of the age-specific exponential decline of normal uEGF values, all analyses regarding uEGF were adjusted for age.

### Genetic Screening

The gene panel used for screening was developed by Dahan et al. (Institut de Pathologie et de Génétique de Gosselies, Belgium) and comprised the following genes: ACTN4, ANLN, APOL1, ARHGDIA, CD151, CD2AP, COL4A3, COL4A4, COL4A5, COQ2, COQ6, COQ8A, COQ8B, CRB2, DGKE, EMP2, GLA, INF2, LAMB2, LMX1B, MAGI2, MYH9, MYO1E, NPHS1, NPHS2, PAX2, PDSS2, PLCE1, PTPRO, TRPC6, TTC21B, WDR73, and WT1.

### Statistical Analysis

Patient characteristics were presented as number and percentages for categorical data, and mean (standard deviation) or median (min–max) otherwise. The relation between categorical characteristics was investigated by Fisher exact test and visualized in mosaic plots.

The log-transformed ratio uEGF/uCreat was studied in a linear mixed effects model with random subject effect and fixed effect age, supplemented with different patient and treatment characteristics and combinations of these. By including a random intercept per patient, dependency among observations of the same individual is taken into account in the analysis. Including all characteristics in one big model was not feasible due to the fact that patient and treatment characteristics are highly entangled; therefore, separate models were fitted to highlight different aspects of the associations.

*Post-hoc* comparisons were made based on these linear mixed effects models, and after back-transformation of the logarithm, results could be expressed as percentage difference, with 95% confidence interval. Bonferroni–Holm correction was applied to adjust for multiple testing.

## Results

### Descriptives

As shown in Table 1, a total of 118 patients were included, of which 62% are boys. From 91 patients 1 to 3 urine samples were collected for measurement of uEGF/uCreat. Median age of this group was 11 years (3–19 years). One hundred and ten patients had undergone a kidney biopsy prior to and independent of this study. In 87, patients a genetic screening had been performed.

**Table 1:** Overview of patients' characteristics, clinical presentation, results of histology, and genetic screening.

	**All patients**		**At least one**	
			**uEGF/uCreat measurement**	
	**Total non-missing**	**Number (%)**	**Total non-missing**	**Number (%)**
**Gender**	118 (100%)		91(100%)	
Male		73/118 (62%)		58/91 (64%)
Female		45/118 (38%)		33/91 (36%)
**Age**	118 (100%)		91 (100%)	
Mean (SD)				11 (4.4)
Median (min–max)				11 (3–19)
**Presentation**	118 (100%)		91 (100%)	
Nephrotic syndrome		75/118 (64%)		54/91 (59%)
Proteinuria		8/118 (7%)		6/91 (7%)
Isolated hematuria		20/118 (18%)		17/91 (19%)
Proteinuria + hematuria		14/118 (12%)		14/91 (15%)
**Biopsy**	110 (93%)		87 (96%)	
Minimal change		45/110 (41%)		31/87 (36%)
FSGS		20/110 (18%)		18/87 (21%)
IgA nephropathy		17/110 (15%)		17/87 (20%)
Alport		20/110 (18%)		15/87 (17%)
Other glomerulopathy		8/110 (7%)		6/87 (7%)
**Genetics**	87 (74%)		69 (76%)	
No genetic cause		45/87 (52%)		37/69 (54%)
Causal mutation		21/87 (24%)		15/69 (22%)
Mutation, unclear significance		21/87 (24%)		17/69 (25%)

*In the left panel, an overview is provided of all patients included into this study. The right panel shows an overview of the patients from whom at least one urine sample was collected and for whom uEGF values are thus available*.

*FSGS, Focal Segmental Glomerulosclerosis; SD, standard deviation; uEGF, urinary epidermal growth factor; uCreat, urinary creatinine*.

The clustered group of “other glomerulopathy” comprised two patients with membranoproliferative lupus nephritis, three subjects with C3 membranonoproliferative glomerulonephritis, one child with diffuse mesangial sclerosis, and one with C1q nephropathy.

### Link Between Initial Presentation and Biopsy Results

Analysis of the 110 patients who had undergone a kidney biopsy, showed a significant link between diagnosis on biopsy and the initial presentation (*p* < 0.001, Fisher exact).

**Figure 1:**
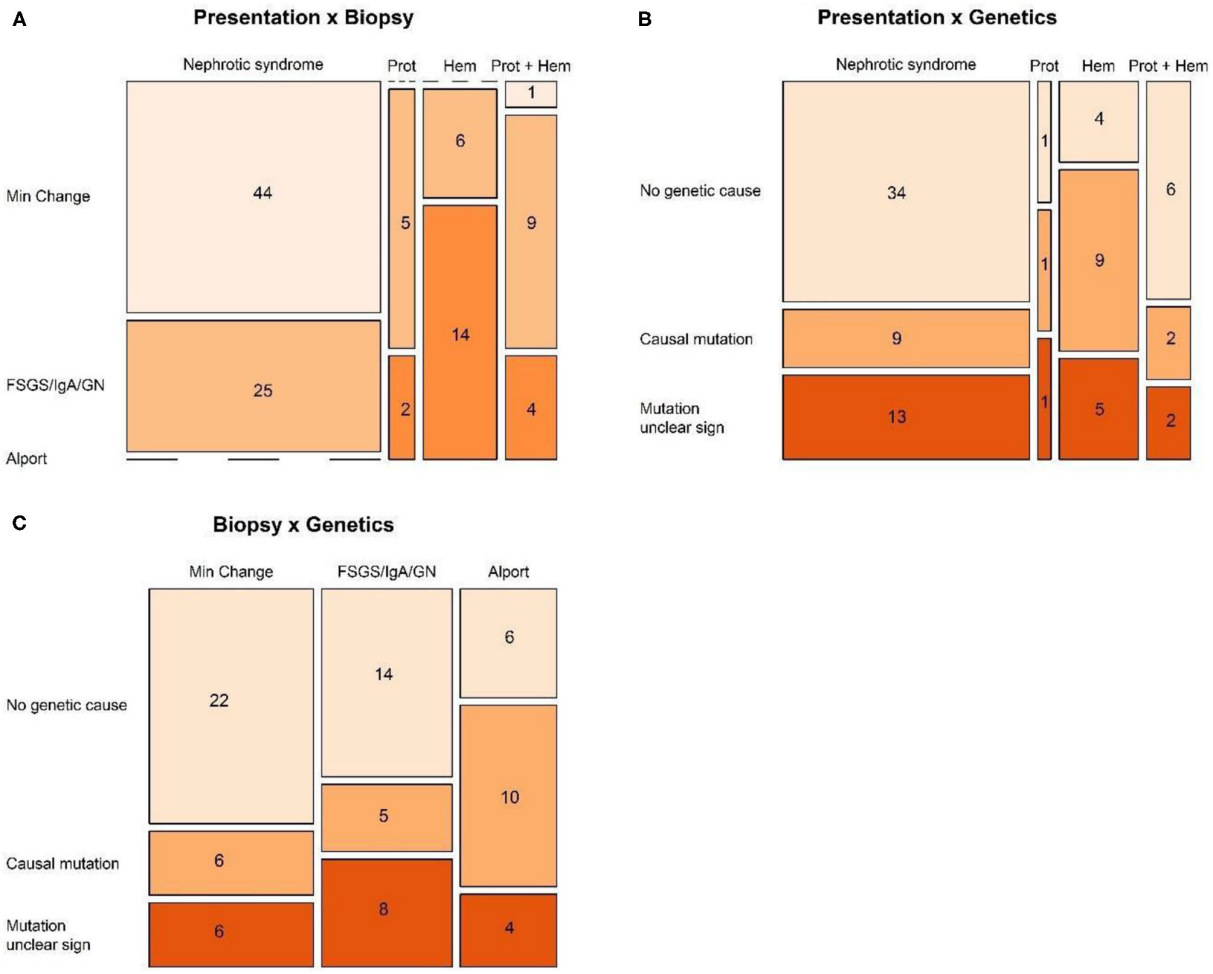
Mosaic plots visualizing the relation between **(A)** presentation and biopsy, **(B)** presentation and genetics, and **(C)** biopsy and genetics.

The diagnosis on biopsy in children with nephrotic syndrome was significantly different compared to the patients displaying non-nephrotic proteinuria (*p* < 0.001). All but one patients with Minimal Change Disease initially presented with nephrotic syndrome. FSGS was only diagnosed in case of nephrotic syndrome, while other glomerulopathies (IgA nephropathy, Lupus membranoproliferative glomerulonephritis, C3 glomerulopathy, etc.) were mostly present in children presenting with non-nephrotic proteinuria, whether or not associated with hematuria, and only in a minority of nephrotic children (Figure 1A).

When disregarding the patients with nephrotic syndrome, and comparing mutually the groups presenting with isolated non-nephrotic proteinuria, combined proteinuria and hematuria, and isolated hematuria, again a significant link was seen with the results on biopsy (*p* = 0.013, Fisher exact) (Figure 1A).

### Link Between Initial Presentation, Genetics, and Biopsy

Analysis of the 87 patients in whom genetic screening had been performed showed a significant link between the genetic result and the initial presentation (*p* = 0.044, Fisher exact), probably a statistical reflection of the fact that in the majority of patients with nephrotic syndrome, genetic screening was not able to show any genetic cause (Figure 1B).

However, there was no significant link between genetic results and diagnosis on biopsy (Figure 1C).

### Link Between Initial Presentation, Biopsy, Genetics, and uEGF

As illustrated in Table 2, patients with nephrotic syndrome had a 46% lower uEGF/uCreat compared to the healthy controls (*p* < 0.001), while no significant difference could be seen for patients with initial non-nephrotic proteinuria (*p* = 0.73). Compared to patients with initial non-nephrotic proteinuria, the subjects with nephrotic syndrome displayed a 32% lower uEGF/uCreat (*p* < 0.001).

**Table 2:** Comparison of uEGF/uCreat between different subgroups.

	**Number of subjects (observations)^b^**	**Difference in uEGF/uCreat (se)**	**Percent diff uEGF/uCreat (95% CI)^c^**	***p*-value**	**Adjusted *p*-value^d^**
**A. Presentation^a^**					
Nephrotic syndrome vs. healthy children	54 (135)	−0.62 (0.097)	−46% (−56 to −35%)	<0.001	<0.001
Proteinuria vs. healthy children	20 (46)	−0.15 (0.13)	−14% (−33 to 11%)	0.24	0.73
**B. Biopsy^a^**					
Minimal change vs. healthy children	31 (79)	−0.57 (0.12)	−44% (−56 to −28%)	<0.001	<0.001
FSGS/IgA/GN vs. healthy children	41 (99)	−0.44 (0.11)	−36% (−48 to −20%)	<0.001	<0.001
Alport vs. healthy children	15 (27)	−0.012 (0.16)	−1% (−28 to 36%)	0.94	1.00
**C. Genetics^a^**					
No genetic cause vs. healthy children	37 (89)	−0.54 (0.11)	−42% (−53 to −28%)	<0.001	<0.001
Causal mutation vs. healthy children	15 (30)	−0.37 (0.15)	−31% (−49 to −6%)	0.017	0.068
Significance unclear vs. healthy children	17 (41)	0.027 (0.14)	3% (−22 to 35%)	0.85	1.00
**D. ACE Inhibitor^a^**					
ACE vs. healthy children	70 (157)	−0.32 (0.10)	−28% (−41 to −11%)	0.002	0.009
No ACE vs. healthy children	21 (53)	−0.66 (0.13)	−49% (−60 to −33%)	<0.001	<0.001

*Comparisons are based on linear mixed effects models with outcome log(uEGF/uCreat), random intercept, fixed effect for age, and one explanatory factor at a time (Presentation, Biopsy, Genetics, and ACE)*.

a *Reference group is healthy children: 49 children, uEGF/uCreat measured once*.

b *Number of patients, between brackets total number of uEGF/uCreat measurements in this subgroup*.

c *Percentage difference compared to healthy*.

d *Bonferroni–Holm adjustment for multiple comparisons (over all comparisons in this table)*.

Additionally, uEGF/uCreat was 42% lower in patients without genetic cause compared to healthy controls (*p* < 0.001) (Table 2C).

### Immunosuppressive Medication and Its Influence on uEGF

Calcineurin-inhibitors (CNI) cause a decrease in uEGF in children (28). As shown in Table 3, this finding was confirmed in our present study, as CNI-treated patients had a significantly lower uEGF/uCreat compared to those patients not receiving CNI (*p* < 0.001). No significant difference in uEGF/uCreat was seen for patients treated with B-cell inhibitory drugs Mycophenolate (MMF) and Rituximab (*p* = 0.27).

**Table 3:** Comparisons based on linear mixed effects models with outcome log(uEGF/uCreat), random intercept, fixed effect for age, Cyclosporine/Tacrolimus, and MMF/Rituximab.

	**Difference in uEGF/uCreat (se)**	**Percent diff uEGF/uCreat (95% CI)**	***p*-value**
**Cyclosporine/Tacrolimus**			
CNI vs. no CNI	−0.44 (0.11)	−36% (−48 to −20%)	<0.001
**MMF/Rituximab**			
MMF/Rituximab vs. no MMF/Rituximab	−0.11 (0.10)	−11% (−27 to 9%)	0.27

*CNI, calcineurin inhibitor*.

When making a distinction based on biopsy results, between minimal change disease on the one hand and every other glomerulopathy identified by aberrant light microscopy and immunofluorescence on the other hand (“FSGS/IgAN/other GN”), no significant difference could be withheld in the number of patients treated with CNIs, or for the use of B-cell inhibitors (Table 4). However, the use of CNI did differ significantly when comparing the nephrotic patients with the non-nephrotic proteinuric group. A difference in uEGF levels between nephrotic and proteinuric patients could therefore be explained merely by this difference in immunosuppressive treatment (Table 5).

**Table 4:** Biopsy results in relation to the treatment regimen.

	**Minimal change *N* = 31**	**FSGS/IgA/other GN *N* = 41**	***p*-value**
Cyclosporine/Tacrolimus	17 (55%)	15 (37%)	0.19
MMF/Rituximab	10 (32%)	18 (44%)	0.45

*In this crosstab, the biopsy results are displayed according to the treatment regimen, hereby providing the absolute numbers and the percentage (%)*.

**Table 5:** Nephrotic patients and non-nephrotic proteinuria in relation to the treatment regimen.

	**Nephrotic syndrome *N* = 54**	**Proteinuria *N* = 20**	***p*-value**
Cyclosporine/Tacrolimus	30 (56%)	2 (10%)	0.001
MMF/Rituximab	18 (33%)	9 (45%)	0.51

*In this crosstab, nephrotic patients and patients with non-nephrotic proteinuria are displayed according to the treatment regimen, hereby providing the absolute numbers and the percentage (%)*.

### Influence of ACE Inhibition on Urinary EGF

A significant beneficial effect of ACE inhibition on urinary excretion of EGF was noticed. Irrespective of the initial presentation or biopsy result, patients receiving ACE inhibition had a 30% higher uEGF/uCreat compared to the patients treated with ACE inhibitors (*p* = 0.007) (Table 6A).

**Table 6:** The effect of ACE inhibition on uEGF/uCreat according to the biopsy diagnosis.

	**Difference uEGF/uCreat (se)**	**Percent diff uEGF/uCreat (95% CI)**	***p*-value**	**Adjusted *p*-value^a^**
**A. ACE vs. No ACE**				
Total: ACE vs. No ACE	0.30 (0.11)	30% (9 to 67%)	0.007	0.044
MCD: ACE vs. No ACE	−0.15 (0.16)	−14% (−37 to 17%)	0.34	0.68
FSGS/IgA/GN: ACE vs. No ACE	0.58 (0.16)	78% (31 to 143%)	<0.001	0.003
**B. Without ACE inhibitor**				
MCD vs. FSGS/IgA/GN	0.45 (0.20)	57% (5 to 133%)	0.026	0.11
MCD vs. Healthy	−0.49 (0.15)	−38% (−54 to −17%)	0.002	0.012
FSGS/IgA/GN vs. Healthy	−0.93 (0.17)	−61% (−72 to −45%)	<0.001	<0.001
**C. With ACE inhibitor**				
MCD vs. FSGS/IgA/GN	−0.28 (0.13)	−24% (−42 to −1%)	0.039	0.12
MCD vs. Alport	−0.66 (0.18)	−48% (−64 to −27%)	<0.001	0.002
FSGS/IgA/GN vs. Alport	−0.38 (0.16)	−32% (−51 to −6%)	0.019	0.096
MCD vs. Healthy	−0.64 (0.13)	−47% (−59 to −31%)	<0.001	<0.001
FSGS/IgA/GN vs. Healthy	−0.36 (0.11)	−30% (−43 to −14%)	0.001	0.008
Alport vs. Healthy	0.026 (0.16)	3% (−25 to 41%)	0.87	0.87

*Comparisons based on linear mixed effects models with outcome log(uEGF/uCreat), random intercept, fixed effect for age, and combination of biopsy result and ACE inhibition*.

a *Bonferroni–Holm adjustment for multiple comparisons (over all comparisons in this table)*.

**Figure 2:**
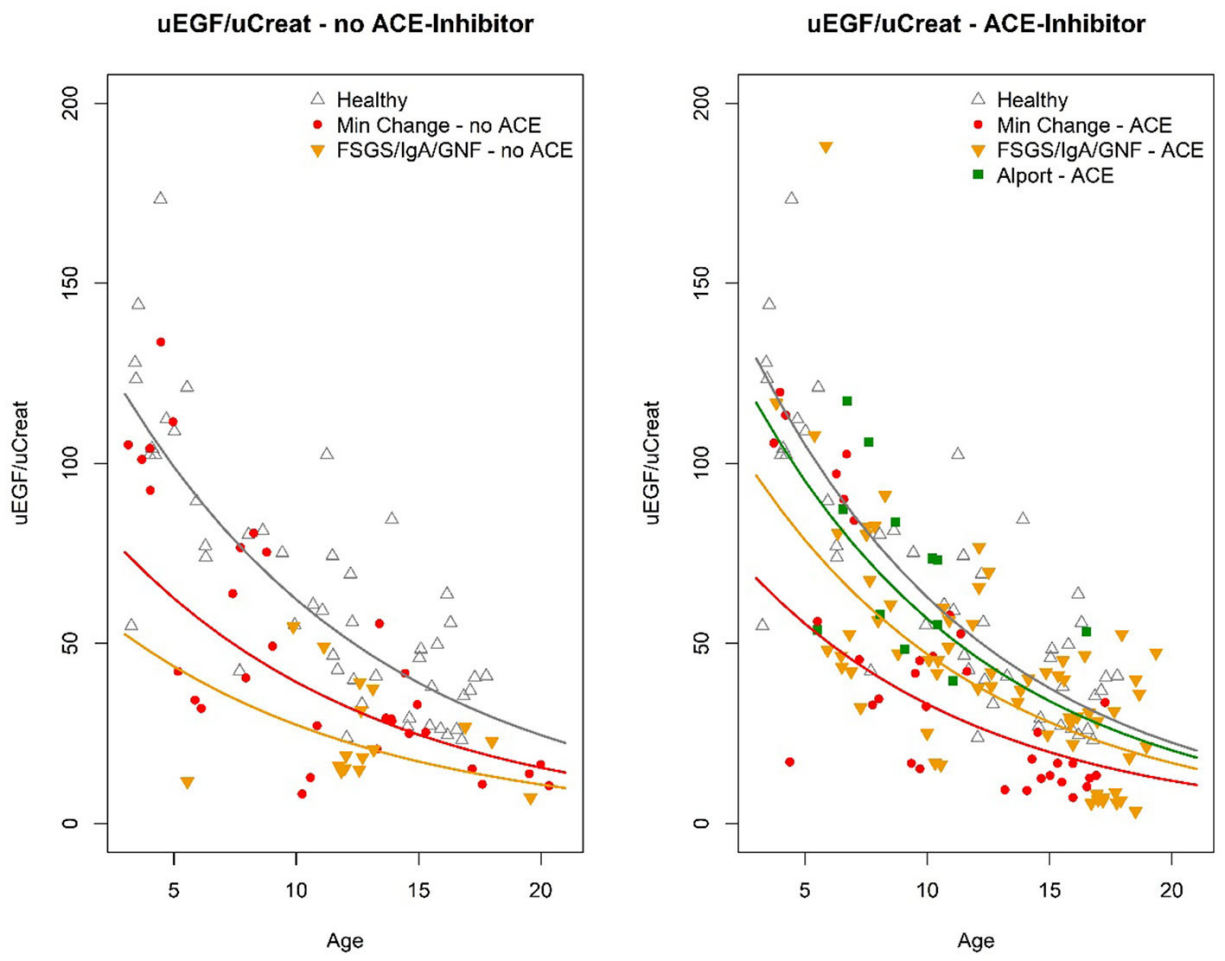
Graphical compilation visualizing the absolute values of U EGF/uCreat for age, according to the different clusters of biopsy results, comparing patients treated with ACE inhibitor to those without ACE inhibition.

To go further into depth on this finding, the biopsy results were subdivided into two types: minimal changes (MCD) vs. obvious glomerular changes visible on light microscopy or immunofluorescence, either FSGS, IgA nephropathy, or any other glomerulopathy (“FSGS/IgAN/other GN”) (Figure 2).

This revealed that the significant beneficial ACE-I effect on uEGF/uCreat was due to a spectacular difference in uEGF/uCreat in the “FSGS/IgAN/GN” biopsy group, where patients treated with ACE inhibitors showed a 78% higher uEGF/uCreat than those without ACE inhibition. ACE inhibition had no significant effect on uEGF/uCreat in the minimal change disease biopsy type (Table 6A).

#### Patients Without ACE Inhibition

Compared to the healthy controls, uEGF/uCreat was significantly lower in both the MCD-group (38% lower than healthy, *p* = 0.002) and in the children with an aberrant “FSGS/IgAN/GN” biopsy type (61% lower than healthy, *p* < 0.001) (Table 6B).

More importantly, a significant difference in uEGF/uCreat was seen between the MCD patients and the patients with “FSGS/IgA/GN.” MCD patients had a 57% higher uEGF/uCreat than the children with FSGS, IgA nephropathy, or another glomerulopathy (*p* = 0.026) (Table 6B).

#### Patients Treated With ACE Inhibition

When focusing on all subjects receiving ACE inhibition, a separate group of Alport patients under ACE inhibition which functioned as a second control showed no significant difference in uEGF/uCreat compared to the healthy controls (Table 6C).

Under ACE inhibition, both in the minimal change disease and in the “FSGS/IgAN/ GN,” a significant difference in uEGF/uCreat was still seen compared to healthy controls as well as in comparison to Alport syndrome (Table 6C).

However, under ACE inhibition, minimal change disease patients no longer had a significantly different uEGF/uCreat compared to the “FSGS/IgAN, GN” group, due to a pronounced increase in uEGF/uCreat in the latter biopsy group.

### Nephrotic Syndrome and uEGF/uCreat

Similar findings resulted from narrowing the focus toward the patients with nephrotic syndrome (Table 7).

**Table 7:** Comparisons based on linear mixed effects models with outcome log(uEGF/uCreat), random intercept, fixed effect for age, and combination of biopsy result and ACE inhibition.

	**Difference uEGF/uCreat (se)**	**Percent diff uEGF/uCreat (95% CI)**	***p*- value**	**Adjusted *p*-value^a^**
**ACE vs. No ACE**				
Total: ACE vs. No ACE	0.16 (0.13)	17% (−9 to 51%)	0.23	0.69
Min Change: ACE vs. No ACE	−0.11 (0.18)	−10% (−36 to 27%)	0.54	1.00
FSGS/IgA/GN: ACE vs. No ACE	0.61 (0.21)	84% (22 to 179%)	0.004	0.021
**ACE inhibitor**				
MCD vs. FSGS/IgA/GN	−0.037 (0.20)	−4% (−35 to 44%)	0.85	1.00
**Without ACE inhibitor**				
MCD vs. FSGS/IgA/GN	0.68 (0.26)	98% (18 to 230%)	0.009	0.039

a *Bonferroni–Holm adjustment for multiple comparisons (over all comparisons in this table)*.

## Discussion

In this study, the urinary profile of EGF excretion was mapped in children presenting with prolonged proteinuria or nephrotic syndrome refractory to or dependent of steroids. We investigated whether, taking into account genetic predisposition, response to immunosuppressive medication and influence of ACE inhibition, uEGF/uCreat could be linked to the underlying biopsy result.

### uEGF/uCreat Is Significantly Lower in Children With a Proteinuric or Nephrotic Glomerular Disease, Despite Preserved Kidney Function

The additive value of uEGF in predicting renal decline in adults and children with progressive kidney disease or with chronic systemic diseases has been elaborated extensively over the 5 years (25, 28, 29). A similar predictive capacity of uEGF regarding renal decline in nephrotic pediatric patients was suggested a year ago by Gipson et al. (19), a multicenter study in which the initial value of uEGF/uCreat in children with minimal change disease or with FSGS was linked to their eGFR.

However, a comparison with a healthy control group was lacking in that study. Normal values of uEGF are very age-dependent (26) and have an exponential decline with age. So, specifically in a pediatric population this major influence of age on urinary excretion of EGF should be reckoned in order to avoid bias by age. Moreover, the impact of immunosuppressive medication or ACE inhibition on uEGF/uCreat was not elaborated either in the study by Gipson et al.

Our present study expands and finetunes the insights published by Gipson et al. We confirmed that children displaying a “difficult to treat” nephrotic syndrome (refractory to or dependent of steroids), but with preserved kidney function, have significantly lower uEGF/uCreat levels compared to healthy controls. As uEGF is a barometer for renal interstitial resilience, this indicates that in our specific pediatric population with normal eGFR, glomerular damage of any degree (even minimal changes) is translated into early secondary interstitial and peritubular changes. The strength of our study compared to the that of Gipson et al. (19) is the fact that we performed repeated measurements of uEGF/uCreat in the majority of the patients (thereby strengthening the consistency of our findings), that the age-specific values of uEGF/uCreat were respected in the statistical analysis, and that these UEGF/uCreat levels were compared with those of healthy controls. Moreover, the impact of immunosuppressive medication and ACE inhibition was extensively elaborated in our study. As we observed a major effect of CNI on uEGF/uCreat, and a pronounced influence of ACE inhibition on uEGF/uCreat, one could wonder if the data described by Gipson et al. would not be a reflection of the use of CNI and ACE inhibition, rather than an intrinsic disease-induced decrease in eGFR.

The fact that also glomerulopathies other than FSGS were included in our study, and displayed a similar pattern of uEGF/uCreat as in FSGS, seems to confirm once more that any immune- imbalance induced glomerular damage (also IgA nephropathy, Lupus nephritis, C3 glomerulonephritis, etc.) has a prompt repercussion on the interstitial “health” of the kidney, irrespective of the underlying pathophysiology causing the glomerular damage, long before a significant decrease in e GFR would be observed.

### Minimal Change Disease Has a Different Urinary Profile of EGF Compared to Other Proteinuric Glomerulopathies: Implications for uEGF as Diagnostic Tool?

The diagnostic and prognostic limitations of a kidney biopsy in children with refractory or steroid-dependent nephrotic syndrome are well-known, since the often-encountered verdict of “Minimal Change Disease” does not evitate the clinical need for strong immunosuppressive treatment, nor does it guarantee less relapses or a better outcome at long term.

In our study, the profile of uEGF/uCreat was significantly different in children with biopsy-proven MCD compared to pediatric patients having any other glomerular diagnosis on kidney biopsy. These findings suggest that uEGF/uCreat could have diagnostic potentials to distinguish minimal change disease from any other glomerular diagnosis, thereby possibly diminishing the indication for a kidney biopsy.

It is reasonable to hypothesize that, compared to MCD, urinary EGF expression decreases more in FSGS, IgAN, and other glomerulopathies due to more pronounced glomerular damage and podocyte dropout. Although our data are perhaps not strong enough to suggest that urinary EGF could completely replace an initial biopsy, it is realistic that monitoring the profile of U EGF over time would help to minimize the need for repeat biopsies. Pairing U EGF/uCreat assessment with biopsy in case of steroid-resistant nephrotic syndrome would, for example, be useful to identify false-negative biopsies that miss the FSGS lesion.

Obviously, to confirm this hypothesis, a study on a larger scale is mandatory, with an expansion of the number of patients as well as with long-term follow-up of UEGF/uCreat in each patient.

### The Role of ACE Inhibition: Emphasizing the Difference Between Minimal Change Disease and Other Glomerulopathies

Urinary EGF is a biomarker for tubulo-interstitial damage and, as such, a hallmark of kidney disease progression, including deterioration of any glomerular disease (17, 18, 23). Besides its direct glomerular effect, ACE inhibition is known to have a major influence on repair and regeneration of peritubular fibrosis secondary to glomerular injury (19, 30, 31).

So, not surprisingly, a significant beneficial effect on uEGF/uCreat was observed in our study population, as uEGF/uCreat was significantly higher in the patients receiving ACE inhibition, irrespective of the initial presentation or biopsy result.

Strikingly however, the rise in uEGF/uCreat was selectively more pronounced in patients with an obvious glomerulopathy on biopsy, compared to children with minimal change disease. Theoretically, this implicates that measuring uEGF/uCreat in nephrotic children before and during ACE inhibition, might help to predict the probability that minimal change disease is the underlying diagnosis on kidney biopsy.

This impressive difference for ACE inhibition reinforces the hypothesis that minimal change disease has a distinct underlying pathophysiology, with less impact of its glomerular damage on the renal interstitial compartment, and as a consequence less sensitivity to ACE inhibition.

Finally, also the observed link between uEGF/uCreat and the absence of genetic predisposition in our study seems to strengthen the idea that, in general, minimal change disease should be considered a separate entity rather than an early precursor of FSGS.

The overall beneficial effect of ACE inhibition in our study population, was also confirmed in the control group of pediatric Alport patients, which under ACE inhibition displayed no significant difference in uEGF/uCreat compared to the healthy controls. Of course, a limitation of this finding was the lack of comparison with Alport patients not receiving ACE inhibition. However, a recent paper on pediatric Alport patients by Li et al. (20) demonstrated that at any age, and irrespective of the presence of proteinuria of kidney function, children with Alport syndrome (not treated with ACE inhibition) display a significantly lower uCreat than healthy controls.

Our study has limitations. The relatively small sample size and the fact that every subject in the study was under immunosuppressive treatment at the time the urine samples were taken are considerable restrictions. Although calcineurin inhibitors are known to influence Ucr, the profile of immunosuppressive treatment in the subgroups of our study did not differ significantly. Also, individual response of uEGF/uCreat to ACE inhibition is lacking, as patients were either already on ACE inhibition at the start of the study, or not receiving ACE inhibition at all during the course of the study.

On the other hand, the fact that all treatment options were taken into account in our study provided extra information on their respective effect on uEGF/uCreat, and even shed a new light on the findings of Gipson et al. (19).

Upgrading the study to a larger scale by increasing the number of patients, would allow to statistically calculate the diagnostic value of uEGF/uCreat in predicting the presence of an underlying minimal change disease.

This study thus opens the road for further investigating the predictive role of uEGF/uCreat in pediatric glomerular diseases, to further unravel their underlying pathophysiology and the mode of action of immunosuppressive drugs, in a larger pediatric population with or without nephrotic range proteinuria.

In conclusion, our study provides several new insights:

1. Despite preserved kidney function, children with a proteinuric or nephrotic glomerular disease on kidney biopsy show a significantly lower uEGF/uCreat, indicative of early tubulo-interstitial damage, which appears partially reversible under ACE inhibition in a degree dependent of the underlying biopsy type.
2. Our data suggest that uEGF/uCreat might be a helpful, non-invasive tool to distinguish minimal change disease from other proteinuric glomerular diseases, by linking the percentage of decrease in uEGF/uCreat to its response on ACE inhibition and to the absence of genetic predisposition.
3. The beneficial effect of ACE inhibition on uEGF/uCreat differs significantly in children with minimal change disease compared to children with obvious signs of glomerular changes on kidney biopsy. This reinforces the hypothesis that a distinct underlying pathophysiology lies at the origin of minimal change disease.

## Data Availability Statement

The original contributions presented in the study are included in the article/supplementary materials, further inquiries can be directed to the corresponding author/s.

## Ethics Statement

The studies involving human participants were reviewed and approved by Ethical Committee of the Antwerp University Hospital. Written informed consent to participate in this study was provided by the participants' legal guardian/next of kin.

## Author Contributions

DT designed the study and wrote the major part of manuscript. NL collected the clinical data into the database and helped writing the manuscript. KW performed all statistical analyses. KL was responsible for the analyses of U EGF by ELISA in the laboratory LEMP. All authors were actively involved in interpreting the results and contributed to the conclusions in the discussion.

## Conflict of Interest

The authors declare that the research was conducted in the absence of any commercial or financial relationships that could be construed as a potential conflict of interest.

## Publisher's Note

All claims expressed in this article are solely those of the authors and do not necessarily represent those of their affiliated organizations, or those of the publisher, the editors and the reviewers. Any product that may be evaluated in this article, or claim that may be made by its manufacturer, is not guaranteed or endorsed by the publisher.

## Abbreviations

MCD: Minimal change disease
FSGS: Focal segmental glomerulosclerosis
EGF: Epidermal growth factor
uEGF: Urinary epidermal growth factor
eGFR: Estimated glomerular filtration rate
ACE: Angiotensin converting enzyme
CNI: Calcineurin inhibitor
MMF: Mycophenolate mofetil
IgAN: IgA nephropathy
GN: Glomerulonephritis
uCreat: Urinary creatinine.
